# Lithium-ion battery electrolyte mobility at nano-confined graphene interfaces

**DOI:** 10.1038/ncomms12693

**Published:** 2016-08-26

**Authors:** Boaz Moeremans, Hsiu-Wei Cheng, Qingyun Hu, Hector F. Garces, Nitin P. Padture, Frank Uwe Renner, Markus Valtiner

**Affiliations:** 1 Institute for Materials Research, Hasselt University, BE-3590 Diepenbeek, Belgium; 2 Max-Planck Institut für Eisenforschung GmbH, 40237 Düsseldorf, Germany; 3 School of Engineering, Brown University, Providence, Rhode Island 02912, USA; 4 IMEC, Division IMOMEC, BE-3590 Diepenbeek, Belgium; 5 Institute for Physical Chemistry, Technische Universität Bergakademie Freiberg, 09599 Freiberg, Germany

## Abstract

Interfaces are essential in electrochemical processes, providing a critical nanoscopic design feature for composite electrodes used in Li-ion batteries. Understanding the structure, wetting and mobility at nano-confined interfaces is important for improving the efficiency and lifetime of electrochemical devices. Here we use a Surface Forces Apparatus to quantify the initial wetting of nanometre-confined graphene, gold and mica surfaces by Li-ion battery electrolytes. Our results indicate preferential wetting of confined graphene in comparison with gold or mica surfaces because of specific interactions of the electrolyte with the graphene surface. In addition, wetting of a confined pore proceeds via a profoundly different mechanism compared with wetting of a macroscopic surface. We further reveal the existence of molecularly layered structures of the confined electrolyte. Nanoscopic confinement of less than 4–5 nm and the presence of water decrease the mobility of the electrolyte. These results suggest a lower limit for the pore diameter in nanostructured electrodes.

## Abstract (web summary)

Electrochemical processes strongly depend on the wetting and mobility phenomena at the involved nano-confined interfaces. Here, the authors use a surface forces apparatus to understand the wetting behavior of graphene, gold, and mica surfaces by Li-ion battery electrolytes.

## Graphical abstract

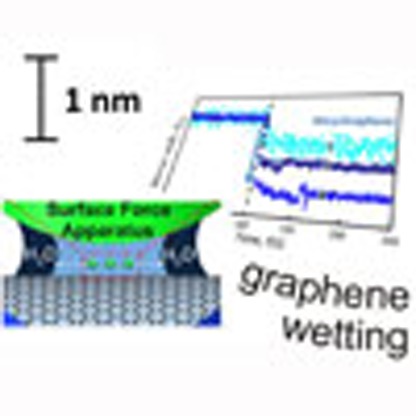

Wetting phenomena at surfaces and interfaces play a crucial role in many natural and synthetic processes, and electrochemical processes are no exception. Recently, nanotechnology advances have triggered an increasing interest in nanoscale engineering for improving power densities in electrochemical devices such as Li-ion batteries. Thus, a detailed understanding of wetting of nano-confined gaps has become essential for making further technological advances in this field^1^,^2^. The wetting and intrusion of pores has been investigated on a microscopic scale in fields ranging from geology^3^, petrochemistry^4^, construction^5^ to modern lithium-ion battery research^6^. In the latter, the nanostructured materials are specifically designed to increase the rate of surface reactions. However, wetting and its related kinetics ultimately depend on the electrolyte homogeneity and its related viscoelastic behaviour within the specific interface volume, with potentially different composition and structure at the subnanometre scale. Thus, in Li-ion batteries, where electrodes typically contain active-material particles, conductive materials and polymer binder, Li-ion intercalation occurs via the interface between the electrode and the electrolyte. For efficient charge transfer processes in nano-porous electrode materials^7^,^8^, it is ideal that the entire surface is wet by the electrolyte. To date, the wettability of electrolytes on electrodes has been studied mainly at a coarse scale by measuring macroscopic properties such as surface tension and contact angle. However, wetting studies at a nanoscale level in general are restricted to molecular dynamics simulations of water uptake in silica nanopores^9^ and Monte Carlo simulations of aqueous electrolytes in nanopores^10^. Apart from modelling intact graphite flake surfaces—the most widely used anode material in commercial Li-ion batteries—graphene itself is being investigated as an important material in the design of novel electrode materials^11^. The addition of graphene to anode materials has been shown to result in superior electrical conductivity, higher surface area, structural flexibility, and thermal and chemical stability^12^.

Here we use multiple-beam interferometry in a Surface Forces Apparatus (SFA) to investigate directly the nanoscale wetting behaviour of a Li-ion battery electrolyte on confined graphene, gold and mica surfaces by a few Å-sized molecular layers of the electrolyte. White-light interferometry in the SFA provides a unique view into the nanolayer structuring of the electrode–electrolyte interface of each electrode material in well-controlled nanoscale-gap geometries. The insights obtained from this study may provide essential understanding of the initial wetting and the guidelines for the design of critical electrode parameters such as nanoscale pore dimensions in terms of mobility of Li-ions at interfaces.

## Results

### Experimental approach

**Figure 1:**
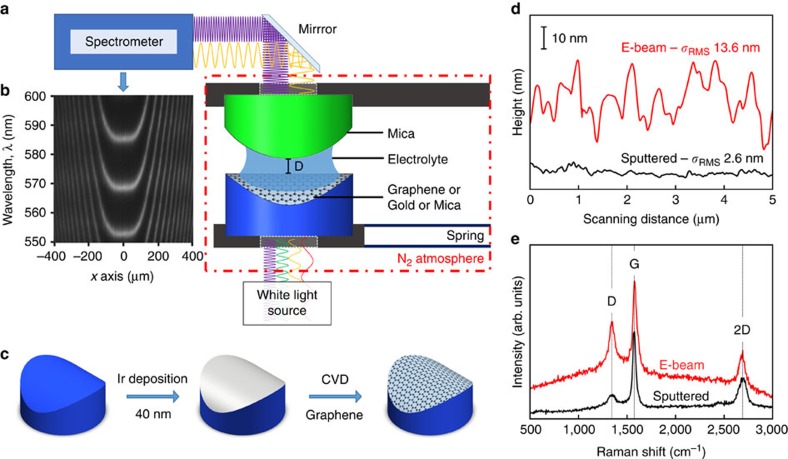
SFA set-up and graphene disk synthesis and analysis. (**a**) Crossed cylinder experimental set-up of the SFA-2000. (**b**) FECO in graphene–mica-opposing materials. (**c**) Schematic illustration of the synthesis of graphene on a silica disk. (**d**) Atomic-force microscopy linescan and analyses of graphene layers deposited on sputtered iridium (black) and on EB-PVD iridium (red) on silica disks, indicating the measured *σ*_RMS_ over areas larger than 25 μm^2^. (**e**) Raman spectra of graphene layers deposited on sputtered iridium (black) and on EB-PVDed iridium (red) on silica disks.

Figure 1a depicts the experimental set-up. In an SFA experiment, a molecularly smooth contact between two opposing materials on crossed cylindrical silica disks is established. The opposing materials are designed in such a way that the two semitransparent opposing mirrors form an interferometer. Here a silver mirror is evaporated on the backside of a transparent mica surface, and the opposing surface is either: (1) also back-silvered mica (symmetric), (2) a gold mirror or (3) an iridium mirror covered with graphene. If white light is guided through such a contact, constructive and destructive interference generates so-called fringes of equal chromatic order (FECO), which can be detected using an imaging spectrometer. The flat part of the FECO indicated in Fig. 1b represents the smooth contact area. The contact area can be manually adjusted to a diameter of typically ∼100 μm. During initial contact formation in dry argon atmosphere, the initial FECO wavelength *λ*_0_, and therewith an absolute zero distance (*D*_MS_=0), is defined. A change in the FECO fringe position (which is recorded as a wavelength shift Δ*λ* away from *λ*_0_) can then be correlated to a shift in distance, Δ*D*, of the opposing disks with sub-Å resolution. As the investigated materials in this study do not swell or contract, this mirror shift allows one to study meticulously the wetting of the opposing investigated surfaces^13^.

Atomically smooth gold surfaces used in this work were prepared by template stripping from mica. Graphene surfaces were synthesized on smooth iridium layers according to a novel protocol shown in Fig. 1c. The graphene-covered disks (Fig. 1c) contain a 40-nm iridium layer underneath, which also serves as a semitransparent mirror. Graphene was grown directly on iridium-covered glass disks using the methane/hydrogen chemical vapour deposition (CVD) synthesis route^14^,^15^. Atomic-force microscopy (AFM) linescans of the graphene layer on the iridium surface deposited using sputter deposition and EB-PVD are shown in Fig. 1d. EB-PVDed iridium yields very rough surfaces, with a *σ*_RMS_=13.6±2 nm. In contrast, a sputtered iridium layer has a very smooth surface with a *σ*_RMS_=2.6±0.6 nm over areas >25 μm^2^, while for areas <25 μm^2^ *σ*_RMS_ is well below 1 nm (see also Supplementary Fig. 1). It is noteworthy that the large-scale roughness level of *σ*_RMS_=2.6 nm originates from the graphene growth process, and it is not from the iridium deposition process itself (*σ*_RMS_<1 nm over >25 μm^2^ before graphene growth). These low *σ*_RMS_ values offer an excellent level of smoothness for SFA studies^16^, and this preparation method will prove useful for many other future SFA experiments. The typical G and two-dimensional (2D) bands in the Raman spectrum of the SFA disks shown in Fig. 1e confirm the presence of graphene on both the EB-PVEDed and sputtered iridium surfaces. The slightly broadened 2D peak indicates double/triple-layer graphene, while the D-band indicates the presence of defects in graphene-deposited iridium. When using the sputtered iridium surface for graphene growth, the very small D-band indicates that the defect density is very low^17^. The high-quality FECO (Fig. 1b) and Raman data indicate an excellent quality of graphene SFA disks prepared using this method compared with direct-transfer methods^18^. In this work similar results with identical conclusions were obtained with both graphene topographies (on EB-PVDed and sputtered iridium), although a higher resolution could be obtained using the smoother graphene (see comparison of FECO from rough and smooth graphene surfaces in Supplementary Fig. 2).

### Nano-confined Li-ion battery electrolyte layering

**Figure 2:**
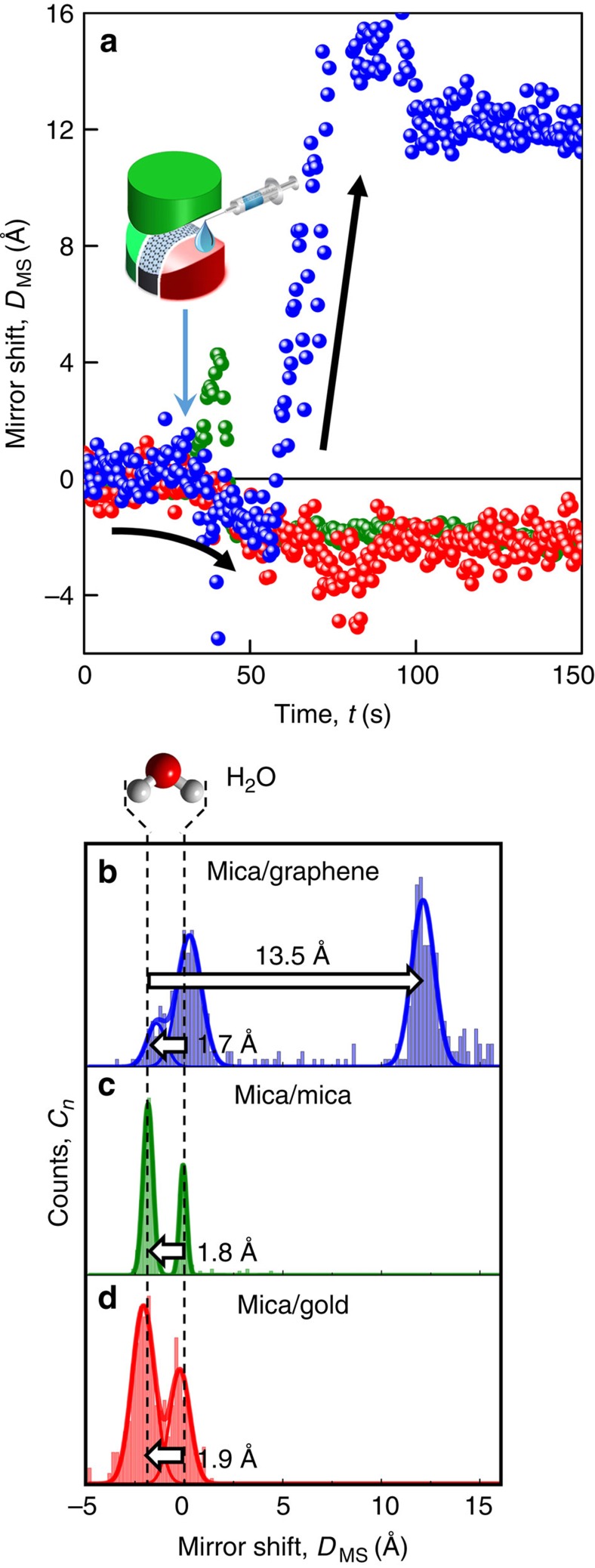
Initial wetting behaviour upon lithium-ion battery electrolyte injection. (**a**) Layer size evolution of the mica–mica (green), mica–gold (red) and mica–graphene (blue) opposing materials during electrolyte injection. Population analysis of the collected data for opposing materials: (**b**) mica–graphene, (**c**) mica–mica and (**d**) mica–gold.

In this work we established a dry initial contact, where opposing surfaces are separated by *D*∼0 nm, and then we injected the electrolyte into the direct vicinity of (1) mica–graphene, (2) mica–gold and (3) mica–mica contacts (Fig. 2) while following the wetting behaviour of the confined interface in real time. Figure 2a shows the initial change in distances just after injecting the electrolyte (1 M LiPF_6_ in ethylene carbonate (EC)/diethylene carbonate (DEC) 1:1 by volume), that is, the wetting behaviour of the investigated opposing materials for a nano-confined interface. As can be seen again in Fig. 2a, the distance between the two disks drops by 1.7–1.9 Å below the initially determined zero-point for all investigated contacts. A population mapping of the mirror shift is plotted in Fig. 2b–d for the three materials used for the lower disk, showing thickness changes during initial wetting. The initial change to negative ∼1.7–1.9 Å in distance can be explained by the extrusion of a monolayer of adsorbed water molecules. We performed our experiments at a relative humidity well below the ambient 40% in an experimental cell that was purged with dry Nitrogen, which leads to a partial monolayer of adsorbed water molecules^19^,^20^,^21^. Upon pressing two mica surfaces together, the partially covered mica surfaces form a monolayer of water. In contrast to aqueous solutions, where the first adsorbed water molecules are immobile because of the high negative surface charge^22^, water is weakly bound and highly mobile at room temperature and low surface charging in the present situation. In addition, Li-ions have a higher affinity to water molecules than to EC/DEC organic solvents in the electrolyte^23^. Thus, in the confined geometry with two opposing surfaces in intimate contact, the weakly surface-bound water molecules are thus drawn out of the contact area into the available Li-containing organic electrolyte. Consequently, the two disks of the set-up will move closer together by one monolayer of water.

As can be further seen in Fig. 2a for mica–mica and mica–gold arrangements, the system remains stable after the initial drop, indicating no wetting of the dried confined area by the electrolyte. The electrolyte is not able to overcome the contact pressure of ∼15–20 bar. In stark contrast, at the mica–graphene interface the distance between the two surfaces increases considerably again after the initial drop, revealing the subsequent formation of a 1.3-nm-thick interfacial film despite the applied pressure. Thus, for graphene, the electrolyte immediately wets the confined area, opening a nano-gap with a width of 1.3 nm. Mechanistically, the occurrence of such material-specific wetting behaviour can be attributed to physicochemical binding interactions between the surface layer and the electrolyte. Possible mechanisms include strong interactions between the organic molecules or Li ad-atoms with graphene.

**Figure 3:**
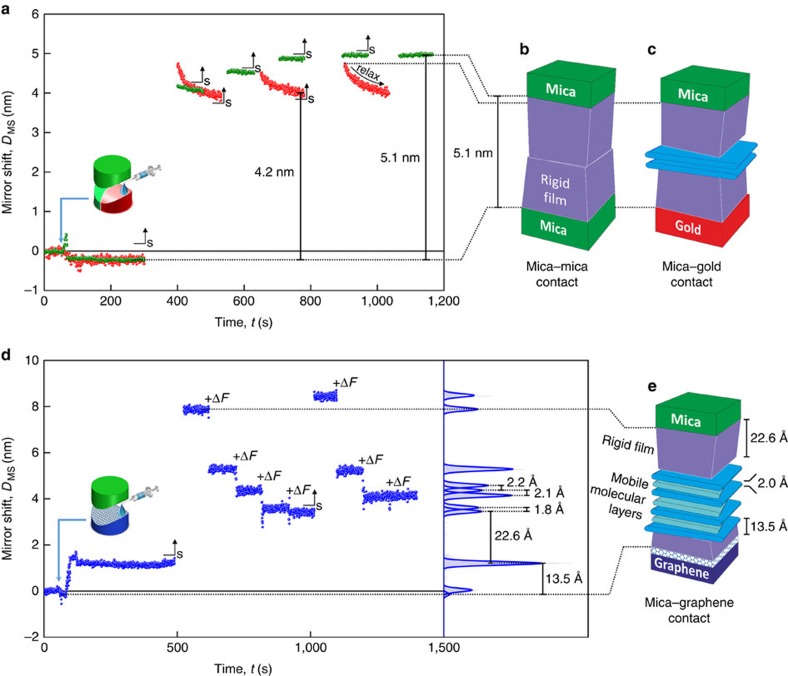
Nano-confined lithium-ion battery electrolyte layering. Layer size evolution for opposing materials: (**a**) mica–mica and mica–gold, and (**d**) mica–graphene. During the measurement, the electrolyte was injected, the surfaces were separated and brought back into contact (_s_), and the force on the spring disk was increased (+Δ*F*). Models of the layer formation in opposing materials: (**b**) mica–mica, (**c**) mica–gold and (**e**) mica–graphene.

In order to study further the mechanical properties of the confined electrolyte, the respective confined SFA disks were separated (*D*>>100 nm) after the initial wetting, allowing the electrolyte to fully penetrate into the contact volume for all interface materials. After a period of 2 min, the disks were brought into close contact again, pushing out most of the electrolyte within the contact. Interestingly, after full wetting of the contacts not all of the electrolyte can be pushed out from all contacts. Figure 3 shows typical multiple approach-retraction (contact-separation) cycles, each marked by an arrow (S). With this simple procedure a more rigid nanoscaled electrolyte film remains on all surfaces, which cannot be squeezed out of the contact area with the pressures that can be reached in the SFA (∼100 bar)^24^. This behaviour indicates that wetting of a confined pore proceeds via a completely different mechanism compared to wetting of a macroscopic surface. In particular, the liquid surface tension in confinement can be considerably different if wetting occurs via a confined pore. This is because of the fact that ions or other species may not necessarily diffuse into an established pore at the bulk concentration, establishing a confined liquid that may differ significantly from the bulk. In contrast, if a macroscopic surface is wet, the liquid surface tension remains at its bulk value and the bulk liquid establishes a macroscopic contact angle.

In the case of a mica–mica arrangement (Fig. 3a, green spheres), the layer size remains stable after the contact is restored and additional pressure is applied, indicating the presence of a rigid electrolyte film between the opposing surfaces. In contrast, in the mica–gold experiment (Fig. 3a, red spheres) the distance slowly relaxes further after initially restoring the confined gap at 4.8 nm. The longer time needed to establish a stable rigid film at 4.0 nm indicates a slower relaxation of the volume of the initial film on the gold surface compared with mica. This more viscous film behaviour may be related to a homogenous film adapting to the confinement over time or near-surface layers, which are initially less ordered. In the mica–mica and mica–gold experiments, no deviation of the behaviour seen in Fig. 3a was observed upon the application of additional force.

In the mica–graphene experiment, increasing the force by increments of ∼5 mN (3.1 bar) results in further stepwise thinning of the confined film thickness, as shown in Fig. 3d. This behaviour can be interpreted as further removal of mobile layers of the electrolyte molecules. The thickness of the interface film is ∼8 nm. However, after a short stabilization period, a further increase in contact pressure can then apparently push out more electrolyte. This indicates an increased mobility of battery electrolytes over graphene surfaces. Here the lowest value of ∼3.6 nm of the total layer thickness can be reached. Assuming a perfect symmetry in the 5.1-nm-thick nano-layer between the two (symmetrical) mica surfaces suggests the thickness of the rigid film on mica to be ∼2.5 nm (Fig. 3b), and hence the rigid-film thicknesses on gold and graphene to be ∼1.6 and ∼1.0 nm, respectively (Fig. 3a, d).

This is very similar to the initial wetting layer on the graphene surface above (∼1.3 nm). The relative changes in thickness with each push-out step vary, depending on the actual distance before the step. During the first pushing steps at wider gaps, that is, larger distances from the lower limit in total thickness (3.6 nm) often larger steps in distance are observed, which we interpret as multiples of quantized single-molecular layers^25^. Indeed, closer to the lower limit in total distance, the jump-ins are consistently 1.8–2.2 Å thick (see also Supplementary Fig. 3). Interestingly, the planar Li–EC solvates Li^+^(EC)_*x*_ (*x*=1, 2 or 3) have a maximum thickness of 1.7 Å (ref. 26). Thus, taking the intermolecular and interlayer electrostatic interactions into account, we speculate that EC-solvated Li ions form the mobile layers in the mica–graphene experiments, explaining also the differences in the mobility at the different interfaces. From all the described results it can be concluded that the mobility of the confined electrolyte layers increases in the order mica<gold<graphene. The flat profile across the mica–graphene interface and a homogeneous mirror shift during the experiments in confinement indicate the rather rigid structure of the films after restoring the contact. All experiments were repeated multiple times, showing similar trends with disks prepared separately. For graphene, the surfaces could be reused showing the same trends in the experiments, proving that no reaction with the electrolyte occurred at defect sites. The role of hydrophobicity on the wetting behaviour was also investigated by growing a hydrophobic 1-undecanethiol self-assembled monolayer (SAM) on a gold disk (Supplementary Fig. 4). The wetting behaviour closely resembles the mica–gold experiment, rather than the more hydrophobic mica–graphene wetting experiment, indicating that graphene surface chemistry, not hydrophobicity, is a crucial factor in the wetting behaviour.

### Influence of water on nano-confined electrolyte

**Figure 4:**
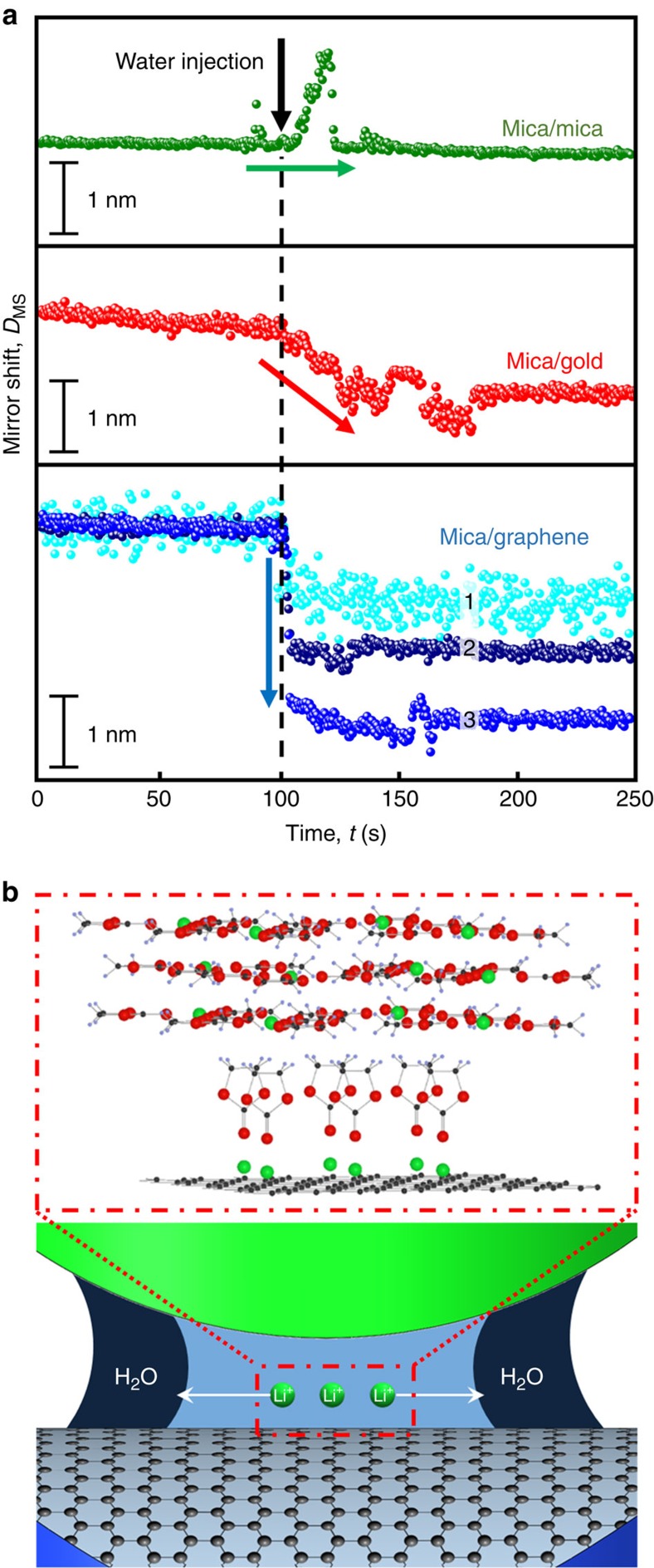
Effect of water on nano-confined lithium-ion battery electrolyte layers. (**a**) Layer size evolution in mica–mica, mica–gold and mica–graphene opposing materials during water injection after electrolyte injection, and multiple contact-opening cycles. Water injections were performed on the mica–graphene contact with increased layer push-out (1>2>3). (**b**) Detail of proposed confined electrolyte layering including mobile Li^+^(EC)_*x*_ layers and schematic illustration of the Li-ion migration towards the water droplet.

It is now also instructive to look directly into the response of nano-confined electrolyte in the presence of water. The presence of even small quantities of water is a nearly unavoidable fact in interfacial processes and, in particular, at Li-ion battery interfaces. Hence, we also injected a small droplet of water into the vicinity of the contact volume, which was first immersed in organic solvent (Fig. 4a), and followed the response of the interfacial layer thickness. Therefore, the SFA disks were contacted at stable pressure by piezo-control after multiple contact-separation cycles.

As can be seen in Fig. 4a, following an initial slow variation of the layer thickness, the confined layer thickness on mica remains stable after water injection. On gold, the initially 4.0-nm-thick wetting layer gradually decreases in layer thickness by 8–10 Å. As such, for both mica and gold this indicates slow material transport over tens of seconds. In contrast, on graphene, as can be seen in Fig. 4a, an abrupt drop within 1 s is observed, indicating very fast removal of interfacial species. For graphene, the water injection was additionally performed at different applied pressures, which resulted in different magnitudes of these sudden drops in the layer thickness. The largest decrease (∼2.5 nm) was observed when no additional pressure was applied after restoring the contact at *D*∼5.2 nm by piezo-control. By increasing the pressure, the confined volume was decreased, and the layer shrinkage was reduced to ∼1.6 and ∼0.9 nm, for initial layer thicknesses of *D*∼4.0 and *D*∼3.4 nm, respectively (see again Fig. 4a). Such a decrease in layer thickness can be attributed to the preferred solvation of interfacial ions by water compared with EC/DEC (visualized in Fig. 4b). Similar to the interfacial water migration during initial electrolyte injection as described above, the motion of the species forming water solvates after the additional water injection is directed away from the confined area. In this case, the Li ions and the respective counter ions migrate from the confined electrolyte layer to the water phase, causing the layer to shrink. Because of their high mobility, ions are able to swiftly migrate to the water phase outside the contact area. As less mobile lithium/EC layers are present when the pressure is increased, the layer contraction decreases with increasing pressures. Such layering shows similar properties to the slip-planes of confined molecular layers of ionic liquids^27^.

The very limited response during the mica–mica experiments is interesting, and may indicate that either no ions are present in this confined geometry, or that ions which are present are very strongly bound to the remaining rigid layer. This may be because of the negatively charged mica surface, which may prevent Li-ions from migrating into the water reservoir to maintain charge neutrality inside the confined zone. The gradual decrease in thickness in the mica–gold experiment fits the model of a more viscous layer, in which solvated ions are present, yet gold may attract considerably higher ratios of EC/DEC compared with ion pairs. The sudden thickness drops in the mica–graphene experiment are consistent with very mobile solvated ion layers and the observed compression behaviour (Figures 3d and 4a). Possible detail of this proposed layered structure on graphene is shown in Fig. 4b with lithium solvates or layered electrolyte molecules present. Directly addressing this three-dimensional interface structure is beyond the scope of the present work and will, because of expected subtle electronic effects, also require support by *ab initio* calculations.

## Discussion

Interestingly, the formation of rigid and mobile interfacial layers suggests a minimum confined thin-film thickness that forms between atomically smooth surfaces without applied external potentials. We expect that the presence of stacked rigid and mobile layers in the direct vicinity of the film is necessary for effective transport and higher diffusion of ion pairs in confined gaps. Thus, for electroactive interfaces^28^, such as electrodes in Li-ion batteries, pores need be wide enough to contain both the rigid layer and the mobile lithium-containing layers for efficient transport of Li-ions in and out of the pores. The exact mechanisms will be very important when manufacturing nanostructured materials, as it can be correlated to the critical pore thickness for a material to be wet by a (re)active electrolyte.

In conclusion, based on the experimental results obtained here we estimate that a graphene-covered pore should have a pore diameter of at least 2–3 nm with the asymmetric opposing materials considered here to ensure the formation of both a rigid inner layer together with a mobile outer layer. Furthermore, ions in confined gold and mica geometries exhibit very limited interfacial mobility below 4–5 nm confinement distance, rendering these materials uninteresting for nanostructuring of electrodes. This limited mobility correlates with the structuring of electrolyte at the respective confining materials. The SFA approach can be used to investigate most interfacial systems to gain better understanding of nanowetting behaviour, nanolayer formation and related kinetic aspects, which may include potential-controlled studies in the future^16^. SFA also allows one to observe directly the initial formation and behaviour of interface layers and their mobility. Beyond the context of Li-ion battery electrodes, the methodology we have developed here is very general as virtually any material can be deposited on the SFA disk with an acceptable RMS roughness. Such insight will have an impact on the targeted design of nanostructured, possibly nanoporous, components in electrochemical devices, where wetting properties and their response to environmental atmospheres (water or other gaseous components), are essential. In particular, our data indicate that wetting of confined pores proceeds via a completely different mechanism compared with wetting of macroscopic surfaces, which have so far been considered as appropriate model systems for understanding nano-scale wetting. Finally, the method for the preparation of graphene-coated silica disks for SFA described here offers a simple and clean transfer-free path to reusable graphene disks that can be useful for many other SFA studies, including adhesion, friction and wear.

## Methods

### Preparation of disks with atomically smooth surfaces

The mica sheets that were used in the experiments were cleaved by hand, producing sheets with uniform thicknesses ranging from 2 to 5 μm and a surface area of typically 5–10 cm^2^. The outer edges of the sheets were melt-cut using a heated platinum wire. The mica sheets were back-silvered using PVD and cut into small pieces, which were subsequently glued with ultraviolet-curable glue (Norland Adhesives, NOA81) on cylindrical silica disks (nominal radius of curvature *R*=2 cm). Atomically smooth gold films were prepared and deposited on SFA disks using a templating technique described elsewhere^29^. The contact reference (*D*_MS_=0) was measured in dry argon between mica and the SFA spring disk (mica, gold or graphene).

An iridium layer with a thickness of 40 nm was deposited on a silica disk using sputter deposition and EB-PVD. Graphene was grown by CVD on the iridium surface in a methane (CH_4_)/hydrogen (H_2_) mixture at 1,000 °C (refs 14, 15). To eliminate impurities during the CVD graphene growth, the iridium surface was annealed at 1,000 °C with H_2_ gas before the introduction of the CH_4_/H_2_ mixture for the graphene formation.

### SFA set-up

Figure 1a shows a detailed schematic diagram of the SFA (SFA-2000, SurForce LLC) set-up used in this study. Two semi-transparent curved disks are placed in a crossed cylinder geometry in a nitrogen atmosphere inside an airtight steel box. The top disk is mechanically fixed. The bottom disk is attached to a spring and displacement mechanism, which allows us to move the opposing disks into a well-defined contact at a given force. The contact is flat because of the glue which is used to fix the mica sheet on the silica disk. During the experiment, white light is guided through these disks, both of which have a semi-transparent mirror: silver on the backside of mica, iridium below the graphene layer and gold on the disk where the metal itself serves as a semi-transparent mirror. When moved close together, these mirrors form an interferometer. The constructive and destructive interference of the white light at discrete wavelengths leads to the generation of the FECO, which are detected by guiding the interfered light into a grating spectrometer using a set of mirrors. A typical FECO is shown in Fig. 1b, which clearly depicts the flat region of fringes, indicating an extended flat contact region. The FECO allows the determination of the intermirror distance with a nominal resolution of 10–30 pm, that is, well below 1 Å. At the same time, the lateral shape of the fringes represents an image of a segment of the contact area, having a lateral resolution of ∼1.0 μm. The flat round-shaped contact area of the mica–graphene set-up, as shown in Fig. 1a, has a diameter of 100 μm. During the initial contact situation in a dry argon atmosphere, the absolute zero distance (*D*_MS_=0) is defined. In the course of the experiment, FECO were recorded at two frames per second and continuously monitored. A change of the FECO fringe position (which is, in fact, a wavelength shift Δ*λ* away from the initial *λ*_0_) can be correlated to a shift in distance, Δ*D*, of the opposing mirrors.

SFA measurements were performed in a cleanroom at 23 °C using the SFA-2000 set-up^13^. The classical way of performing a force-run was not possible in these experiments because of the strong layering and limited range of motors (even with very stiff springs). The rigid layer formation could only be studied by applying very high pressures using micrometre-driven approaches. A force run in aqueous electrolytes with the graphene disks is shown in Supplementary Fig. 5, indicating general suitability of the prepared disks.

### Li-ion battery electrolyte

The electrolyte used was LiPF_6_ (1 M) dissolved in a mixture of EC and DEC in a 1:1 volume ratio.

### Raman spectroscopy

A confocal Raman microscope (WITec Alpha 300) was used to obtain Raman spectra. A Nd:YAG 532 nm laser was used as the excitation source, with a holographic grating of 600 grooves/mm (BLZ 500 nm) and a 50 μm diameter pinhole. The laser power incident on the surface sample was kept low in order to avoid decomposition and damage of the formed graphene. The Raman spectrum in Fig. 1e represents the average of a 10 μm linescan across the entire area.

### Atomic-force microscopy

AFM (MFP-3D Origin, Oxford Instruments) in the tapping mode with 268 scan lines and 268 scan points with a scan rate of 0.10 Hz was used to capture the topography of the graphene layer over a large area (Fig. 1d).

### Data availability

All relevant data are available from the authors upon request.

## Additional information

**How to cite this article:** Moeremans, B. *et al*. Lithium-ion battery electrolyte mobility at nano-confined graphene interfaces. *Nat. Commun.* 7:12693 doi: 10.1038/ncomms12693 (2016).

## Supplementary Material

Supplementary Information Supplementary Figures 1-5.
