# Supplementary material for: Lithium-ion battery electrolyte mobility at nano-confined graphene interfaces
Source: Nat Commun. 2016 Aug 26;7:12693. doi: 10.1038/ncomms12693 (PMC5007463; doi:10.1038/ncomms12693)
Supplement: Supplementary Information — Supplementary Figures 1-5. [file ncomms12693-s1.pdf]

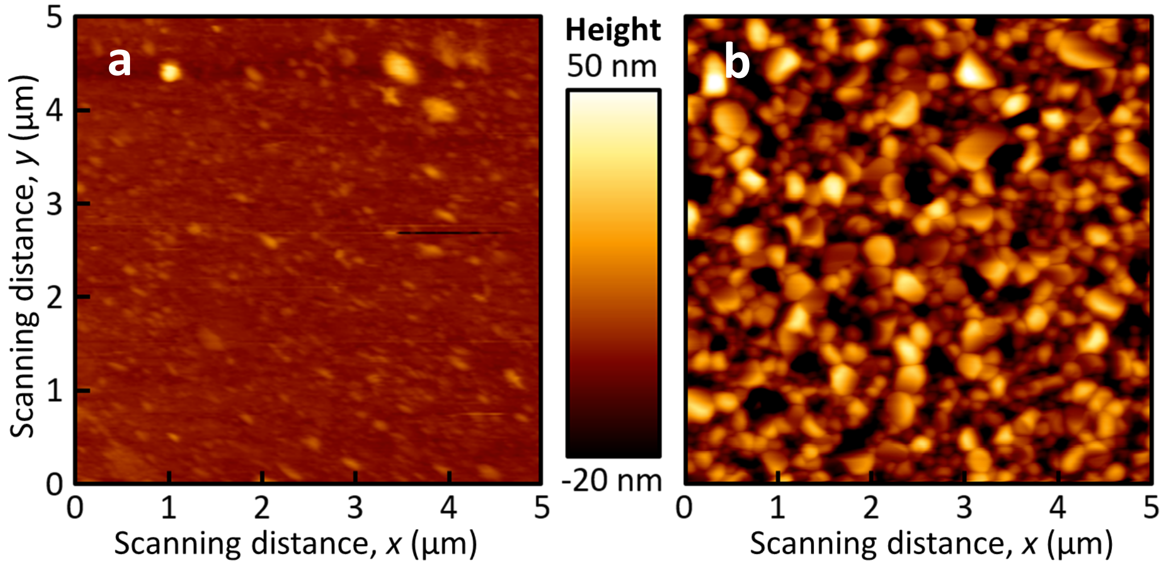

Supplementary Figure 1. Atomic-force microscopy 2D analysis of graphene layer grown on a) sputtered iridium layer and b) E-beamed iridium layer, both on SiO_2 disk. a) has a root mean square roughness of 2.6 nm over the whole area, and less than 1 nm on small areas (0.5 µm x 0.5 nm).

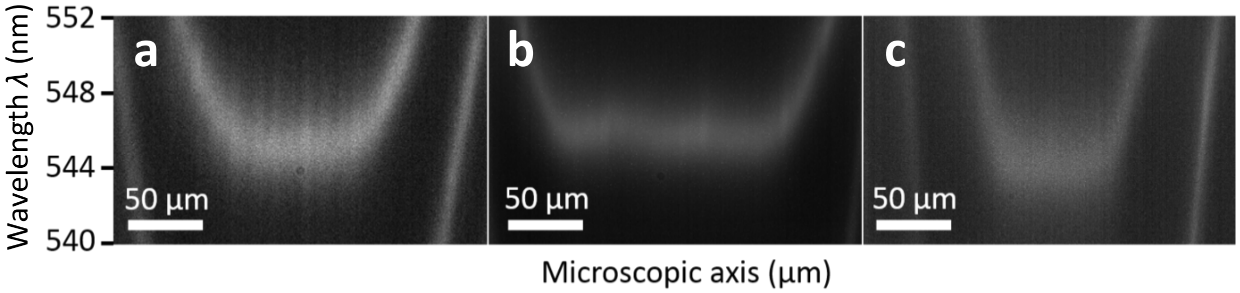

Supplementary Figure 2. Fringes of Equal Chromatic Order (FECO) of graphene deposited on (a) E-beamed Ir and (b-c) sputtered Ir. In a) a lot of small peaks are visible, confirming the roughness observed in Atomic-force microscopy. In b) only 2 peaks are observable, most likely originating from dust particles. c) depicts a very smooth contact spot.

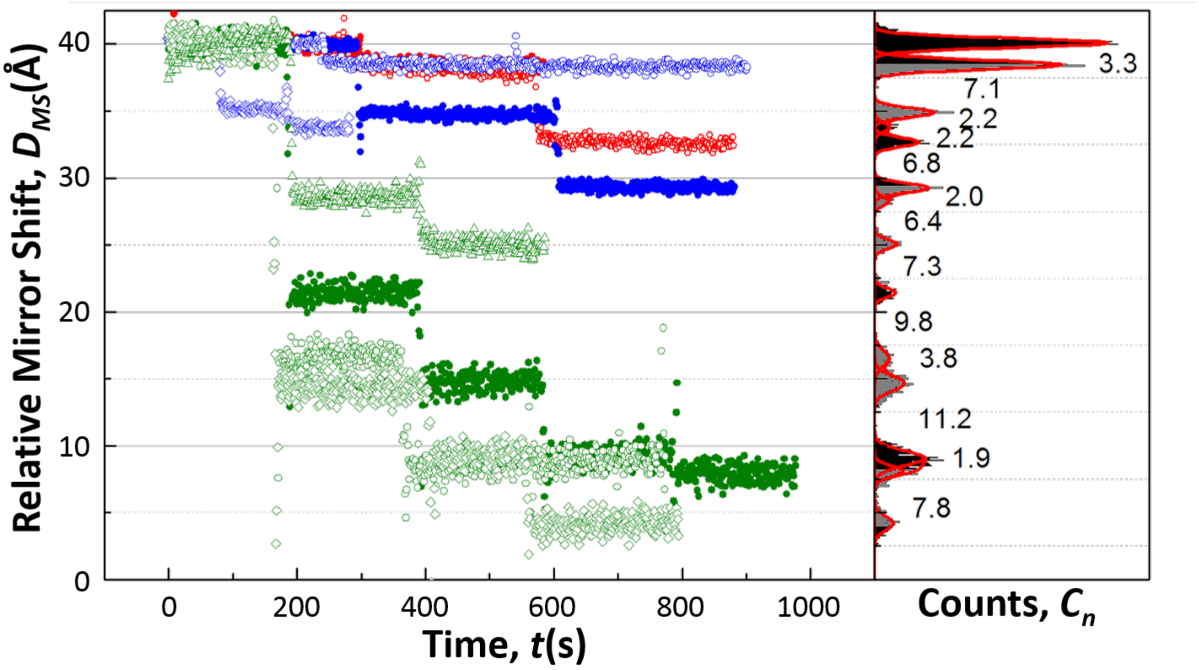

Supplementary Figure 3. Layer size evolution of mica-graphene. The plot shows the decrease in layer thickness by increasing the force on the spring disk after opening and closing the contact. Population analysis of a large set of data on mica-graphene shows that the smallest step is around 2Å.

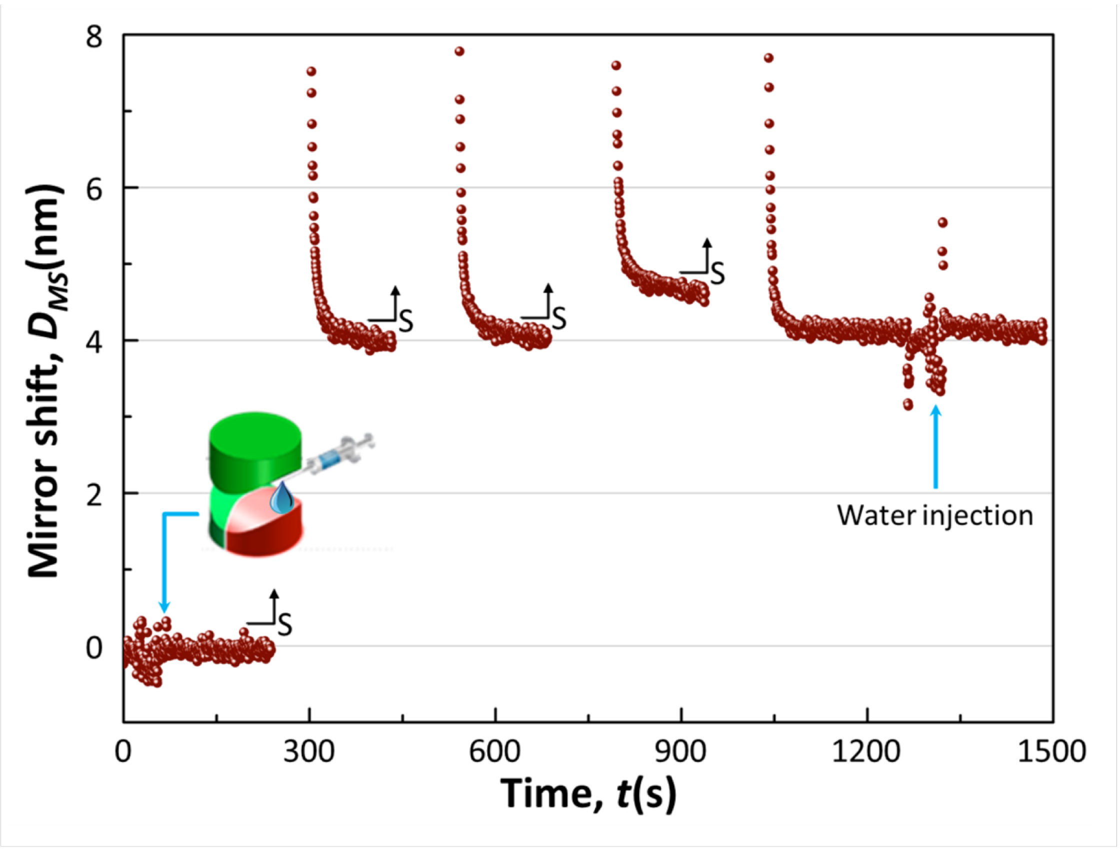

Supplementary Figure 4. Layer size evolution for confined contact of mica and self-assembled monolayer on atomically smooth gold. During the measurement, the electrolyte was injected, the surfaces were separated, brought back into contact (↲S) and finally water was injected in the vicinity of the contact. Relaxation after restoring the contact is faster compared to the mica-gold experiment. As the relaxation process is complete before the water injection, no mobile lithium-ions are able to move out of the contact, leaving the layer thickness unchanged.

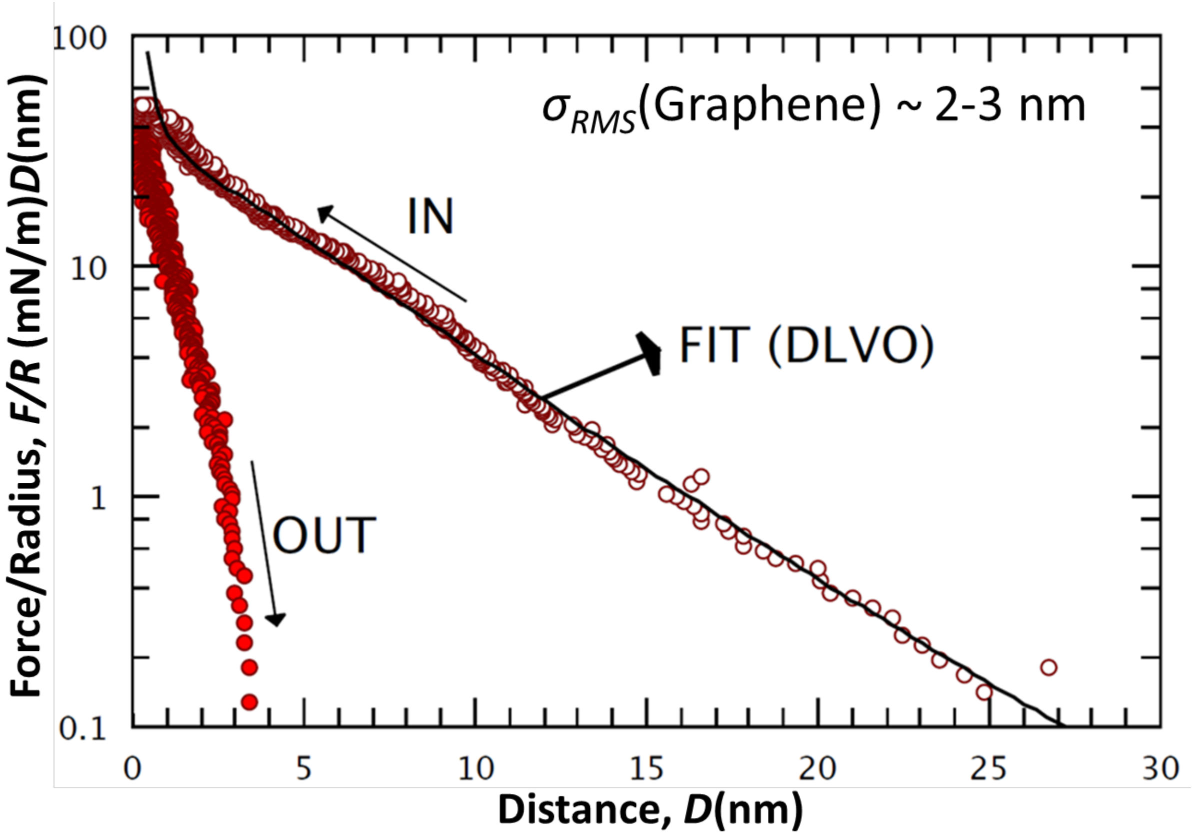

Supplementary Figure 5. Force run across a contact of apposing graphene and mica surfaces in 5 mM NaCl solutions. The approach curves can be fit well using DLVO theory and an additional contribution for the observed roughness. The roughness shows an exponential behaviour as function of the distance with a decay length of 2-3 nm, which is directly related to the RMS-roughness. Interestingly, during separation the observed strong adhesive minimum of F_{ADH} = 5 mN/m is located at D ~ 3nm between mica and graphene-covered Iridium, suggesting either a compression of asperities below D < 3nm or a bridging of the surfaces by the graphene layer between the surfaces. In any case this data demonstrates the quality of the developed graphene surfaces for both wetting as well as surface force studies.
